# Microstructure and Mechanical Properties of Polyacrylonitrile Precursor Fiber with Dry and Wet Drawing Process

**DOI:** 10.3390/polym13101613

**Published:** 2021-05-17

**Authors:** Hyunchul Ahn, Jae-Hyung Wee, Yong Min Kim, Woong-Ryeol Yu, Sang-Young Yeo

**Affiliations:** 1 Advanced Textile R&D Department, Korea Institute of Industrial Technology, 143 Hanggaulro, Sangnok-gu, Ansan-si 15588, Gyeonggi-do, Korea; hahn@kitech.re.kr (H.A.); jhwee0506@kitech.re.kr (J.-H.W.); 2 Department of Materials Science and Engineering (MSE) and Research Institute of Advanced Materials (RIAM), Seoul National University, Seoul 08826, Korea; kymins@snu.ac.kr (Y.M.K.); woongryu@snu.ac.kr (W.-R.Y.)

**Keywords:** polyacrylonitrile, precursor, drawing, microstructure, mechanical properties

## Abstract

Polyacrylonitrile (PAN) fibers are typically used as precursor fibers for carbon fiber production, produced through wet-spinning processes. The drawing process of the spun fiber can be classified into dry and wet drawing processes. It is known that the drawing stability and stretching ratio differ depending on the drawing process; however, the elementary characteristics are approximately similar. In this study, the mechanical properties of PAN fibers have been examined based on these two drawing processes with the differences analyzed through the analysis of microstructures. Further, to examine the composition of the fiber, element analysis has been conducted, and thereafter, the microstructure of the fiber is examined through X-ray diffraction analysis. Finally, the characteristics of PAN fibers and its mechanical properties has been examined according to each drawing condition. There are differences in moisture content and microstructure according to the drawing process, and it affects the tensile behavior. The results obtained could have potential implications if the processes are combined, as it could result in a design for a stable and highly efficient drawing process.

## 1. Introduction

Polyacrylonitrile (PAN) fibers are typically used as a precursor of carbon fibers, with more than 90% of the total carbon fiber produced being based on them [1]. PAN fiber has several advantages for manufacturing carbon fibers such as high carbon yield and stable structure. Moreover, it is suitable for mass production, and has been researched and developed in various ways over decades and also applied commercially [2,3]. The PAN-based precursor fibers are primarily manufactured by wet spinning process, including dry-jet wet spinning, and is the most researched among all carbon fiber precursors, with the aim to improve the physical properties because the mechanical properties of precursors have a significant influence on the mechanical properties of carbon fibers [4]. In particular, for carbon fiber production, it is essential to stabilize the PAN fiber for oxidation and carbonization process as well as for mass production with high quality and uniformity. Consequently, for the improvement of mechanical properties and manufacturing process, many studies have been conducted to improve physical properties and process stability by controlling the copolymers and molecular weight of PAN [5]. For PAN copolymers, the use of methyl acrylate (MA), methacrylic acid (MAA), and itaconic acid (IA) to increase the spinnability and process stability has been studied [6]. Recently, MA and IA have been used occasionally because of their high efficiency through various combinations and polymerization [7]. Typically, they can improve the stability of the post-spinning process efficiently with a content of less than 2% [8]. Furthermore, studies have been conducted to increase the molecular weight of PAN or increase the concentration of dope to improve the mechanical properties of PAN fibers [9,10]. For example, Morris et al. manufactured high-performance fibers by using more than 1.5 million g/mol molecular weight PAN polymers [11]. In the case of high molecular weight as well as concentration dope, the fiber spinning process was performed through high pressure or additional processes because high viscosity hinders the process [12]. Therefore, to overcome this, dry-jet wet spinning can be employed, which secures spinnability with viscous dope and controls the temperature of dope and coagulation bath separately. Thus, a study on the manufacturing process of high molecular weight fibers has been conducted using dry-jet wet spinning process [13].

However, the spun PAN fiber requires a washing and drawing process for use as a fiber or a precursor, and the PAN fiber is typically stretched 10–30 times to finally prepare a precursor fiber [14]. The drawing process is a process that affects structure and has been studied as a post-processing process [15]. The strength of fibers was affected by the stretching [16], and the physical properties of fibers were changed according to solvents and temperature [17]. However, most studies on the extension of wet spinning-based PAN precursor fibers have been conducted as a part of the spinning process based on wet drawing that follow the wet spinning process [18,19]. Subsequently, the spun fiber drawing process can be divided into dry and wet methods, where the dry method is relatively easy to control temperature, but there are still certain limitations in controlling the atmosphere and temperature change between steps. In contrast, the wet method has uniform heat transfer and stable drawing process; however, there are limitations in temperature due to the diffusion and boiling of solvent such as water, and further, an additional drying or post-treatment process is required. Thus, in commercial processes, improved wet processes that removes residual solvent and secures process stability are extensively used, such as a high-pressure steam process that overcome the above mentioned drawbacks [20]. Although the drawing process is a standardized process and it has less impact on the mechanical properties than the spinning process due to its processing limitation, and primarily impacts its reliability. However, micro-structural changes due to the drawing process affect the stabilization and carbonization process in the further carbon fiber manufacturing, which may affect the mechanical properties of carbon fibers [21]. Nevertheless, there is not much research on the difference or structural change in the drawing method. Recently, an alternative method using infrared radiation (IR) has been researched and developed to study the process efficiency and behavior in the drawing stretching process; however, because it is a stretching process that has a physicochemical limitation, it is primarily improvement if process efficiency rather than being a new process [22]. In contrast, for non-contact processes such as IR, stretching and stabilization can be performed at one step; thus, integration studies using them are also being conducted [23].

**Figure 1:**
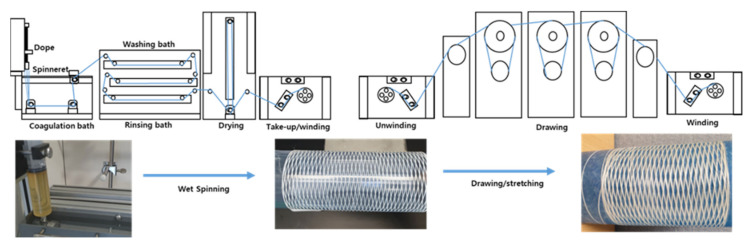
Schematic representation of the process of wet-spun PAN fiber.

In this study, for the analysis of the PAN-based fiber spinning process, the microstructural change of the copolymer PAN-based precursor fiber was investigated based on the drawing process. For the fiber spinning, the material used was prepared by polymerizing a PAN copolymer. Subsequently, changes in mechanical properties and microstructure based on the drawing process were examined for the fibers manufactured through dry/wet stretching conditions (Figure 1). First, the residual solvent was checked through elemental analysis of the material and the as-spun fiber and, thereafter, the drawn fiber was analyzed to confirm whether humidification occurred according to the drawing process. Furthermore, for the microstructure analysis, structural changes according to the drawing process, such as crystallinity and pore structure, were analyzed through two-dimensional wide-angle X-ray diffraction (WAXD) and small-angle X-ray scattering (SAXS). Thus, the effect of the drawing process on the microstructure and the change in mechanical properties were accordingly examined.

## 2. Materials and Methods

### 2.1. Materials

The polymerization of the PAN copolymer was conducted through the typically used basic solution polymerization [11,24]. The materials used were acrylonitrile (AN, Sigma-Aldrich, St. Louis, MO, USA), methyl acrylate (MA, Junsei, Tokyo, Japan), and itaconic acid (IA, Acros Organics, Geel, Belgium), and finally dimethyl sulfoxide (DMSO, Alfa Aesar, Ward Hill, MA, USA) was used as a solvent for polymerization. Thereafter, the polymerization was performed using a,a’-azobis-isobutyronitile (AIBN, Junsei, Tokyo, Japan) and 1-dodecanethiol (Sigma-Aldrich, St. Louis, MO, USA) as an initiator and chain transfer agent, respectively. The composition of the copolymer was prepared as AN:MA:IA = 93:6:1 and the molecular weight of the prepared PAN copolymer was measured to be 139,600 g/mol. Further, in the wet spinning process, a dope was prepared with a DMSO (Dajung, Siheung, Korea) solvent; thereafter, the polymerized PAN was washed to remove the solvent and moisture, and then dried at 100 °C for 6 h. Furthermore, in the coagulation and washing baths, DMSO and distilled water were used.

### 2.2. Fiber Spinning and Drawing

**Figure 2:**
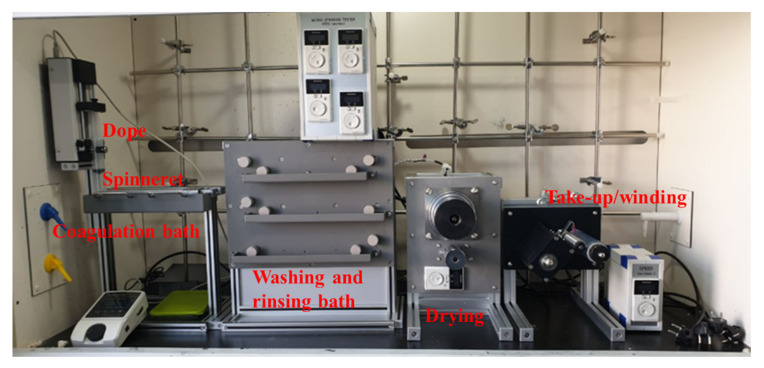
Lab-scale wet spinning equipment and spinning process.

For fiber spinning, a lab scale wet spinning system as shown in Figure 2 was used, consisting of a syringe pump with multi-hole nozzle, coagulation bath, drawing and washing bath, dryer, and winding. The dope was designed to extrude a uniform amount through a syringe pump, and spinning was performed through nozzles with 100 circular holes each having a diameter of 100 μm. The dope spinning condition was 0.2 mL/min with 25 wt.% concentration, and for the coagulation bath, the weight ratio of DMSO and water was 5:5 at room temperature. Thereafter, the PAN fibers formed in the coagulation bath were washed in three washing baths for fiber formation. For each condition, the first bath was filled at 5:5 ratio of DMSO and water, while the second and the third baths consisted of only water. Moreover, all were maintained at room temperature. Subsequently, the fiber after being washed with water was wound through a simple dryer without any additional stretching process involved. However, to maintain the tension in the process, minimal stretching was performed, and approximately 50% of the overall stretching occurred during the process between the coagulation bath and winder. Following the winding, the fiber was immersed in distilled water for an additional 48 h to discharge the residual solvent, and subsequently dried and stored at room temperature and humidity.

**Figure 3:**
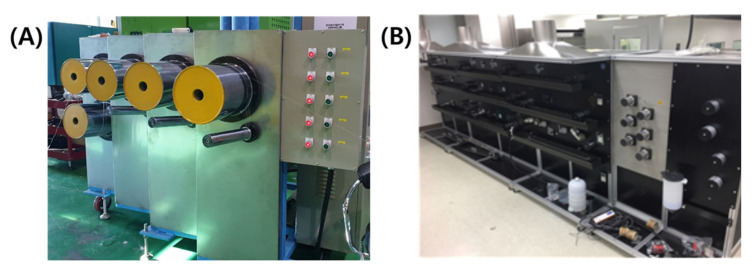
Drawing equipment (**A**) dry drawing, and (**B**) wet drawing.

The spun PAN fiber was stretched 10 times through a dry or wet drawing process, and the stretching ratio was uniformly stretched at a slightly lower value than that of the actual carbon fiber precursor manufacturing process, such that the drawing process could be compared without other factors. Wet-spun PAN precursor fibers stretched 15–25 times generally. In comparison, this study used about half of the drawing ratio. The drawing equipment is shown in Figure 3, where for dry drawing, 1000% of the drawing was performed over three stages at 130 °C after preheating at 70 °C. The wet drawing conditions were preheated in the first bath, followed by multistage stretching from the second bath onwards, and the temperature of each bath was 80, 85, 85, 90, 90, 90 °C. Thereafter, each fiber was naturally dried without any post-treatment after stretching and stored at room temperature. As mentioned before, in the spinning process, 50% of natural stretching was performed to maintain the tension in the washing, drying, and winding processes, and thus, the final fiber was stretched by 1500%.

### 2.3. Characterization

Elemental analyzer (EA, EA2000, Thermo Fisher Scientific, Waltham, MA, USA) analysis was performed to confirm residual moisture and solvent in the material, and the residual solvents were identified through elemental analysis of carbon (C), hydrogen (H), nitrogen (N), sulfur (S) and oxygen (O). The EA analysis was conducted twice to measure each element, where C, H, N, S analysis was performed at 1000 °C with a tungstic anhydride catalyst, while O elemental analysis was performed in the same equipment with nickel plated carbon as a catalyst and at 1060 °C. Subsequently, the residual amount of DMSO as a solvent was confirmed by checking the residual S content of the material as well as the spun fiber, while the residual moisture in the drawn fiber was confirmed through the O content analysis of the spun fiber. Further, the surface structure and spinnability of the fiber were observed through electron microscopy (FE-SEM, SU8000, Hitachi, Tokyo, Japan), and the fiber diameter was measured based on the microscope image obtained. Consequently, the microstructure of the fiber was measured via wide-angle X-ray diffraction (WAXD) (D8 Discover, Bruker, Billerica, MA, USA), with a radiation wavelength of 0.154 nm (Cu Kα). Measurement of microstructures such as crystallinity in fibers through WAXD has been conducted in many studies [25,26]. In addition, measurement of the orientation of crystals using 2D WAXD patterns has also been conducted [27]. In a similar manner, 2D WAXD was measured for the fiber to analyze the microstructure in the fiber. Further, the pore structure analysis in the fiber was measured using small-angle X-ray scattering (SAXS) (Xeuss2.0, Xenocs, Grenoble, France), at a wavelength of 0.154 nm (Cu Kα). Finally, the linear density and mechanical properties of the fibers were measured using a single fiber tester (FAVIMAT, Textechno, Mönchengladbach, Germany), and measurements were performed at 20 mm/min at a 20 mm gauge length, and tensile tests were conducted in each of the 20 or more experiments.

## 3. Results and Discussion

### 3.1. Elemental Composition

The results of elemental analysis of each fiber and material are shown in Table 1, with the corresponding elemental analysis measuring C, H, O, and S elements, and the remaining parts being N. As a result of the material analysis before spinning, S was detected, which is a residual solvent (DMSO) remaining in the polymerization process that is distributed among PAN molecules and is not removed during the washing process. Based on the element fraction, it appears that 2.08% of DMSO remained within the washed and dried PAN material, while the undrawn yarn, which had undergone additional residual solvent removal after fiber spinning, was measured again. In contrast, in the spinning process, though solvent used to prepare the dope was also DMSO, it was confirmed that the residual solvent was removed through a washing process using distilled water in a water bath after spinning. However, fiber composition after each drawing process with the undrawn fiber, yielded no significant difference, but it was evident that the amount of O changes, which is due to the change in residual moisture in the fiber during the wet process in the current drawing and drying process. In particular, because drying at room temperature and humidity was performed for comparison based on the stretching conditions after stretching, when wet drawing is performed, it was not removed from the inside of the fiber, and thus the content of O is high due to residual moisture.

### 3.2. Microstructures

**Figure 4:**
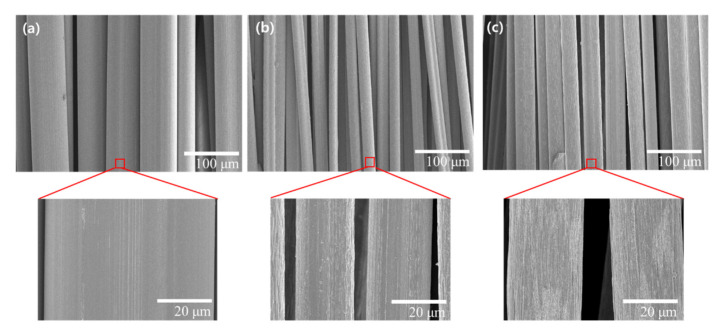
Scanning electron microscopy (SEM) images for surface structure of wet-spun fibers. (**a**) as-spun, (**b**) dry drawing, and (**c**) wet drawing.

The surface of the spun fiber is shown in Figure 4, and the measured diameter and standard deviation of the spun fiber for the as-spun, dry drawing and wet drawing fibers are 65.5 (2.2), 20.7 (1.4), and 22.7 (1.2) μm, respectively. Thus, a uniform fiber was spun and in the case of dry drawing, it was confirmed that the cross-sectional area decreased by approximately 10 times, similar to the drawing ratio, and it was also confirmed that a larger pattern in the drawing direction occurred on the enlarged surface. In contrast, for wet-drawn fiber, it is evident that the cross-sectional area is approximately 10% larger than that of the dry-drawn fiber, and the surface of the fiber is also a little rougher. Due to this difference, it can be confirmed that the process of the same draw ratio or difference in density and cross-sectional area appears. As a result, residual moisture should be removed in the stretching process to form dense precursor fibers. Thus, the dry process can form fibers of a denser structure through efficient removal of moisture at a stable stretching ratio.

**Figure 5:**
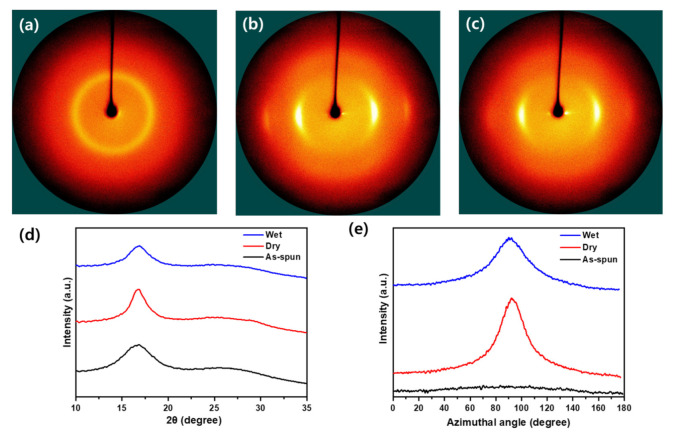
Two-dimensional wide-angle X-ray diffraction (WAXD) images of general wet-spun PAN fibers. (**a**) as-spun, (**b**) dry drawing, (**c**) wet drawing fiber, (**d**) 1D equatorially extracted XRD plot, and (**e**) Azimuthal scans at 2θ = 17°.

In addition, to examine the microstructure, the fiber was examined through WAXD and SAXS. The crystal structure and orientation in the fiber can be investigated using 2D WAXD equatorially and azimuthal scans. The measurement results for 2D WAXD are shown in Figure 5. When considering the equatorially extracted one-dimensional XRD plot, a strong and weak peak appear in all fibers (Figure 5d). The strong peak appears at 2θ = 17°, which indicates (100), while the weak peak at 2θ = 27° is not clearly evident in this result, it indicates (110) [27,28]. *Crystallinity* can be calculated by Hinrichsen’s method (Equation (1)), where *A_c_* is the sum of the areas of peaks and *A_a_* is the sum of the amorphous areas in one-dimensional XRD plot [26,29].

(1) $$Crystallinty=\frac{A_{c}}{A_{a}+A_{c}}$$

(2) $$L_{c}=\frac{K\lambda }{\beta cos\theta }$$

Crystal size (L_c_) can be calculated by Equation (2), where K is a Scherrer parameter having a value of 0.89 for half-widths, the wavelength (λ) used in the device is 0.154 nm, and β is the full width at half maximum (FWHM) of the strong peak [30,31].

The analysis results are shown in Table 2, and the crystallinity of the dry-drawn fibers show a higher result than wet-drawn fibers, which appears to be the effect of the removal of residual moisture. In the elemental analysis, it is evident that the crystallinity of the wet-drawn fiber, which had a relatively high moisture content, decreased the crystallinity, and the degree of orientation was also lower than that of the dry-drawn fiber. When considering crystal size, it is evident that the difference is 2 times for dry-drawn fiber compared to undrawn fiber and 1.5 times for the wet-drawn fiber. This is because the crystals are arranged and deformed in the stretching direction. When the orientation was changed from 6.75 to over 80%, the deformation was thought to have occurred in the stretching direction based on the drawing process rather than the growth of the crystal.

**Figure 6:**
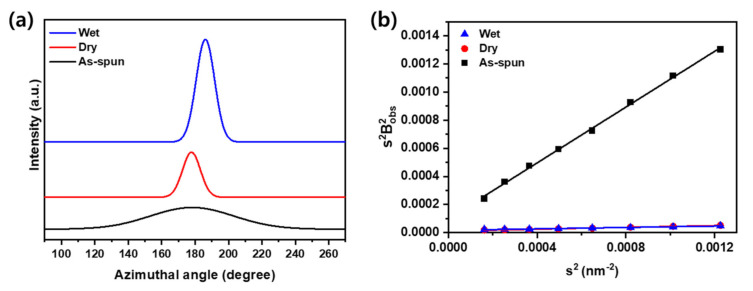
Two-dimensional small-angle X-ray scattering (SAXS) results of PAN fibers. (**a**) Azimuthal scan of the fibers and (**b**) Ruland streak linear fitting.

From the perspective of microstructure, PAN fibers have voids in the fibers when they are manufactured due to the characteristics of the wet spinning process owing to the exchange and diffusion of solvents and non-solvents in the coagulation process, and this can be measured through 2D SAXS [26,32]. The shape of the void in the fiber is similar to a circular shape in the coagulation process, but it changes into a needle shape through the stretching process [28]. Accordingly, the void structure in the fiber is determined by the void length and void angle depending on the process, similar to the WAXD analysis. Furthermore, the void structure is one of the important variables that determine the properties of a fiber as a defect in the fiber. Thus, to analyze this effect, the void structure was analyzed using the Ruland Streaks method of azimuthal scan data in various *s* (*s* is the scattering vector) in 2D SAXS (Figure 6), and the calculation method is briefly described as follows [32]. The integral breadth *B_obs_*(*s*) is obtained by fitting the azimuthal distribution with the Gaussian function expressed as

(3) $$s=\;\left|s\right|\;=2\sin\theta /\lambda$$

where θ is the Bragg angle and λ is the wavelength of the incident X-ray beam (0.154 nm). Thereafter, the integral breadth *B_obs_*(*s*) of each azimuthal scan was calculated using Equation (4)

(4) $$B_{obs}\left(s\right)\;=\frac{1}{I\left(s,\;\pi /2\right)}\int I\left(s,\phi \right)d\phi$$

where ϕ is the azimuthal angle, *I*(s, *π*/2) is the peak height in the azimuthal scan at *π*/2 for a particular *s*, and $\int I\left(s,\phi \right)d\phi$ is the area of the peak in the azimuthal scan.

(5) $$s^{2}B_{obs\;}^{2}\left(s\right)\;=1/L_{c}^{2}+s^{2}B_{eq}^{2}$$

where *L_c_* is the average void length and *B_eq_* is the average void angle.

Thus, the integral breadth and the corresponding value of s^2^ were calculated. Further, through linear fitting, the void angle (B_eq_) in slope and void length (L_c_) in y-intercept can be obtained. Table 3 shows the void structure analyzed using the Ruland streaks method. As seen in the WAXD analysis, in this study, based on the relatively low temperature and draw ratio, it is believed that there is no substantial change in the volume of crystals and pores, and the change in size is due to the deformation and orientation change. In particular, because the shape of the pores is easier than the crystal, it is considered that large deformation close to the needle occurs freely, and the result also indicates a larger change than the change in crystal size. Further, the pore size shows a change of 280% in the case of dry stretching and 235% for wet stretching. In contrast to the undrawn yarn, which can be randomly oriented at a void angle close to 45°, the drawn yarn has an orientation of approximately 10°. In addition, it is evident that the voids are deformed according to the stretching and are oriented in the drawing direction, and it is thought that the voids changed to a sharper shape due to a larger deformation in the relatively dense dry-stretched fibers. However, wet drawn fibers have better void orientation because of their relatively high moisture content and low density than dry drawn fibers, so that the interaction between molecules is greater because of the low resistance to the arrangement. As a result, the shape of the void appears to be sharper in dry stretching and more oriented in wet stretching, and it is believed that a combination of these result in a design for a stable and optimized stretching process.

### 3.3. Mechanical Properties

**Figure 7:**
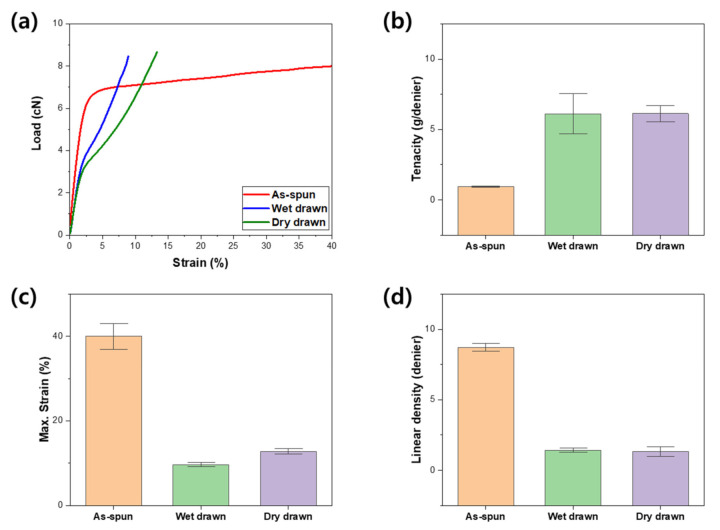
Mechanical behavior of PAN fibers. (**a**) Representative load-strain behavior, (**b**) tenacity, (**c**) maximum strain, and (**d**) linear density of the fibers.

The mechanical properties of the fabricated fibers are shown in Figure 7, and no significant difference between the dry and wet drawn fibers is observed. The strength of a single fiber is not significantly different depending on the stretching, and when stretching is performed, the cross-sectional area decreases; thus, the specific strength increases relatively. This is evident from the tenacity of Figure 7b, and it shows that the difference in stiffness between the dry and wet drawn fibers is similar, but there is a difference in tensile behavior. As mentioned previously, in the case of the wet-drawn fibers, the cross-sectional area was a little larger at the same draw ratio, but the density decreased; therefore, the linear density of the fibers as shown in Figure 7d is similar to that of the dry-drawn fibers. However, as mentioned in the previous analysis results, for wet-drawn fibers, relatively more water molecules exist inside, which appears to cause an early breakdown through the interaction of water molecules [28]. The uniform distribution of water molecules has been studied for having a positive role in the strength of fibers, and appears to play a similar role because the water molecules remain inside after both dry and wet drawing [28]. In this study, an additional drying process other than natural drying at room temperature and humidity was not performed after the drawing process, specifically for the wet drawn fibers. This may have been a factor in the deterioration of strength because wet drawn fibers contain more than the amount of water molecules required for strength improvement. Due to this minor difference in the amount and distribution of water molecules in the fiber, the strength of wet drawn fibers is slightly lower than that of dry drawn fibers. Thus, the strength difference according to the drawing process is small, but there is a structural difference due to the excess water molecules. Therefore, the dry drawn fiber has higher elongation due to its stable and dense structure, while for the wet drawn fibers wherein the water molecule interacts with the PAN molecules in the fiber has higher modulus and rigidity. The PAN precursor fibers according to the drawing process have different behavior due to the microstructure difference, which can be used for further process design. Specifically, in the stabilization process where the elongation affects the stability of the process, the stiffness is slightly lower under the same drawing ratio, but the dry drawn fiber with good elongation appears to aid in the process optimization. In the optimal mechanical property after post-treatment, the fiber using the wet drawing process appears to be more advantageous in structural optimization, which will be helpful when water molecules content is controlled.

## 4. Conclusions

In this study, the microstructure changes of spun fibers were examined based on their drawing process using synthesized PAN copolymers. To investigate the changes in the microstructure of fibers after stretching, the effects of residual solvents were excluded by confirming the removal of solvents through EA analysis of materials and undrawn fibers. The microstructure analysis was conducted through WAXD and SAXS, and the crystallinity of fibers according to the stretching was examined. Further, the effect of dry and wet drawing was compared and analyzed at the same drawing ratio. Through this, the structural changes of pores were investigated according to the drawing methods, and it was determined that it also affects the mechanical properties of fibers. The effect of this is expected to have an impact on the carbonization and graphite process in the carbon fiber manufacturing process and, further, it is expected to optimize the process through comparative analysis. Furthermore, a high efficiency drawing process design is expected if the two processes are combined.

## Figures and Tables

**Table 1:** Element analysis results of each fiber.

	C (%)	H (%)	S (%)	O (%)
PAN powder	65.759	5.579	1.091	3.954
As-spun fiber	65.901	5.623	0	1.605
Dry drawing	66.197	5.554	0	1.152
Wet drawing	66.260	5.590	0	2.041

**Table 2:** Microstructural characterization results of PAN fibers.

	As-Spun	Dry Drawing	Wet Drawing
Crystallinity (%)	66.66	69.51	65.21
Preferred Orientation (%)	6.75	85.52	80.45
Crystal size (nm)	21.55	42.05	31.64

**Table 3:** Void structure of PAN fibers.

	As-Spun	Dry Drawing	Wet Drawing
Void length (nm)	100.08	284.33	236.16
Void angle (°)	57.06	10.24	8.89
